# Preparation and Characterization of Calcium-Incorporated *Rosa roxburghii* Tratt and Its Efficacy on Bone Mineral Density in Rats

**DOI:** 10.1155/2022/5122396

**Published:** 2022-04-20

**Authors:** Yanfang Yan, Zhongsheng Luo, Leilei He, Fuxiao Wei, Ming Gao, Xiaofang Cui, Juan Yang, Yichun Sun, Lilang Li, Lishou Yang, Tingfei Deng, Xiong Pan, Mei Peng, Yunfei Tan, Zhengbin An, Liangqun Li, Xiaosheng Yang

**Affiliations:** ^1^State Key Laboratory of Functions and Applications of Medicinal Plants, Guizhou Medical University, Guiyang 550014, China; ^2^The Key Laboratory of Chemistry for Natural Products of Guizhou Province, Chinese Academy of Sciences, Guiyang 550014, China; ^3^Guizhou Cancer Hospital, Guiyang 550008, China; ^4^Sinopharm Group Tongjitang (Guizhou) Pharmaceutical Co., Ltd., Guiyang 550009, China

## Abstract

The deficiency of traditional calcium preparation will gradually be replaced by the new type of calcium preparation. *Rosa roxburghii* fruit (*R. roxburghii*) is popular for its rich nutrients and functional ingredients. The fermentation broth of *R. roxburghii*, involving amino acids, flavonoids, triterpenes, polysaccharides, and other compounds, is favorable for calcium chelation. Thus, this study fabricated calcium-incorporated *R. roxburghii* (FECa) and further illustrated its efficacy on bone mineral density (BMD) in rats. The calcium holding capacity of FECa was identified and confirmed using AAS. Ion complexation of FECa was characterized using ^1^H-NMR, UV, SEM and EDS, and FTIR. The calcium contents of femurs were increased by 36%, and the bone trabeculae of femurs were significantly increased. Net calcium balance was enhanced to further improve BMD by oral administration of FECa. The above results indicate that FECa can be a potential and efficient calcium supplementation agent.

## 1. Introduction

It is well recognized that calcium plays an important role in mammals. Calcium, accounting for approximately 1.5–2.2% of body weight, is a necessary major element in the human body. Approximately, 99% of calcium is in the bone and plays a key role in maintaining bone strength [1, 2]. Moreover, a wide range of biological functions of calcium have been found, such as muscle contraction and nerve impulse transmission [3]. However, calcium deficiency has already become a worldwide nutritional deficiency public health problem [4]. An appropriate amount intake of calcium is indispensable for human health. Recent studies have shown that the concentration of intracellular calcium ions can affect osteoporosis, involving proliferating osteoblasts and chondrocytes. Its mechanism is to promote mineralization and bone formation by activating CaV 1.2 L-type voltage-gated Ca^2+^ induces calcium ion influx, raising the intracellular calcium ion level [5, 6]. Osteoporosis is a metabolic bone disease that can induce calcium loss, and over 200 million people have been suffering from this disease [7, 8]. Efficacy in preventing steroid-induced osteonecrosis of traditional Chinese medicine (TCM) and ethnic medicine-derived herbs or compounds in rats has been reported [9]. For example, Xian Ling Gu Bao Fufang, a famous herbal ethnic medicine formula capsule of Guizhou, has been approved by the China Food and Drug Administration (China, Z20025337) [10].

*Rosa roxburghii* Tratt (*R. roxburghii*), belonging to the Rosaceae family, has high edible and medicinal value as a source of homologous medicine and food [11]. *R. roxburghii* is popular for its rich nutrients and functional ingredients. According to the record of the “Guizhou Tong Zhi,” the fruit of *R. roxburghii* has been historically used for 380 years as an ancient Chinese medicine for the treatment of different diseases [12]. *R. roxburghii* fruit is consumed in the clinic to strengthen the spleen and exert antidiarrheal and astringent effects. Accounting for its bioactive functional factors, *R. roxburghii* fruit has been widely applied and is one of the bulk resources in Guizhou, China; meanwhile, fermentation processing is one of the worth options for long-term storage and utilization of its fruit [13]. Products of fermented fruit have been included in the human diet and have been the most fundamental food; these foods also exhibit many healing properties and could prevent many diseases [14, 15].

The fermentation broth of *R. roxburghii,* containing amino acids, flavonoids, triterpenes, polysaccharides and other compounds, is favorable for calcium chelation, according to our preliminary experiment [16]. The deficiency of traditional calcium preparation will gradually be replaced by the new types of calcium preparation. Due to the good solubility and body absorption of organic calcium, this study fabricated calcium-incorporated FECa and further illustrated its efficacy on bone mineral density (BMD) in rats. Thus, this study optimized the preparation of calcium-incorporated *R. roxburghii* (FECa) to further illustrate its characterization and evaluate the efficacy on bone mineral density (BMD) in low-calcium diet-fed SD rats. This work supports the innovative use of *R. roxburghii* for calcium supplement.

## 2. Materials and Methods

### 2.1. Materials

Chromatographic grade methanol and acetonitrile were purchased from Merck (Darmstadt, Germany). Other reagents used were of analytical grade, and guaranteed reagents were all supplied by Tianjin Kemiou Chemical Reagent Co., Ltd. (Tianjin, China). Standard solutions were prepared from 1000 mg/l calcium single-element standard solution (National Center of Analysis and Testing for Nonferrous Metals and Electronic Materials, Beijing, China, GSB 04-1720-2004).

### 2.2. Preparation of FE and FECa

*R. roxburghii* Tratt was collected from Guizhou Province, and fresh fruit was collected in September 2019 (voucher specimen number: CL201901). All samples were authenticated by Prof. Qingwen Sun, Guizhou University of Traditional Chinese Medicine. Jiu Qu (commercially available and containing yeasts and enzymes in the specification) was purchased from Guizhou Brewing Engineering Technology Development Center, Guizhou Keli Industrial Co., Ltd. (Guiyang, China) (batch number: q-qk05-2018).

One kilogram of fresh *R. roxburghii* Tratt fruits was washed and cut into small pieces (thickness of approximately 0.1 cm). Twice the amount of sterilized distilled water was added to beat for one minute by a homogenizer. *R. roxburghii* Tratt fruit pulp was treated with 0.5% Jiu Qu and 0.2% pectinase along with adding 10% white sugar according to the slurry quality into a sterilized triangular flask and incubated at a temperature of 35°C in a shaker incubator at 120 rpm for 20 days.

FECa was made, and the procedures mainly involved two main steps, including the reaction of *R. roxburghii* Tratt fruit fermentation (FE) and CaCO_3_ and the removal of CaCO_3_. Different amounts of CaCO_3,_ including 0.2002 g, 0.3005 g, 0.4010 g, 0.5009 g, and 1.0013 g CaCO_3,_ were added to FE liquid (20 mL) and reacted for 24 h. The mixture was centrifuged at 5000∼ 6000 rpm for 10 min, and the supernatant was collected. The FECa and FE dryness samples were obtained by freeze-drying.

### 2.3. Determination of Calcium Holding Capacity

The calcium content of the FECa sample was determined by flame atomic absorption spectrometry (Agilent, 240FS-240Z, Agilent Technologies Inc., CA, USA) to determine the calcium holding capacity of FECa.

### 2.4. Characterization of FECa

The surface morphology of the FECa and FE samples was observed by scanning electron microscopy (SEM, ZEISS Gemini 300, USA). Dried samples of FECa and FE were coated with gold to improve their electrical conductivity and thermal stability. SEM observation was performed under the condition of accelerating voltage at 25 kV with a magnification of 200× and 10000×. The surface chemical composition of FECa and FE was examined by energy dispersive spectroscopy (EDS, JEM-2100, JEOL, Ltd., Japan).

### 2.5. FTIR, ^1^H-NMR, and UV Spectroscopy

A Fourier-transform infrared (FTIR) spectrometer (iCAN9, Tianjin spectroscopy Technology Co., Ltd., China) was used to record the FTIR spectra of FE, FECa, CaCO_3,_ and FE + CaCO_3_. The background spectrum of the KBr pellet was subtracted from the FTIR spectra. Dried samples of FE and FECa mixed with KBr (1 : 200) were ground and pressed into a 1 mm pellet. FTIR analysis was conducted in the frequency range from 400 to 4000 cm^−1^ with transmission mode at a resolution of 4 cm^−1^.

^1^H-NMR spectra of FE and FECa samples were recorded on a Bruker spectrometer (Bruker AMX600 WB, Switzerland). FE and FECa samples were prepared by dissolving the product (25 mg) in 1 ml deuterated water (99.99%).

UV spectra of FECa and FE solutions (2 mg/mL^−1^) were recorded by spectroscopy on a Cary-60 spectrometer (Agilent Technologies Inc., CA, USA) in the wavelength range of 190 to 600 nm.

### 2.6. Evaluation of FECa on BMD in SD Rats

#### 2.6.1. Animals and Animal Treatment

For all experiments, 4-week-old male Sprague–Dawley (SD) rats weighing 60–80 g (purchased from SPF (Beijing) Biotechnology Co., Ltd., SCXK (Beijing) 2021–0009) were used. According to the international rules for animal experiments and guidelines for the appropriate use of experimental animals (NIH publication no. 80–23; revised 1978). The acclimatization at a temperature of 23 ± 2°C in the SD rats was performed for at least 1 week before the experiments with free access to water and standard food. All the study protocols were performed according to the international rules for animal experiments and approved by the Animal Ethics Committee of Guizhou Medical University (clearance no. 2000882).

Seventy SD rats were randomly divided into 7 groups (*n* = 10 in each group): normal control group (NC), model control group (MC), low (L), middle (M), high (H), CaCO_3_ group, and calcium gluconate group (CG). The NC group and MC group were administered 0.5% sodium carboxymethyl cellulose, L was administered FECa equal to 20 mg/kg calcium, M was administered FECa equal to 40 mg/kg calcium, H was administered FECa equal to 60 mg/kg calcium, the CaCO_3_ group was administered CaCO_3_ equal to 40 mg/kg calcium suspended with 0.5% sodium carboxymethyl cellulose, and the CG group was administered calcium gluconate equal to 40 mg/kg calcium. The NC group was fed a normal diet, and the L, M, H, CaCO_3_ and CG groups were administered a low-calcium diet (calcium content ≤150 mg/kg). All the rats were treated with gavage for 28 days. Before sacrifice, all SD rats were individually placed into metabolic cages for collection of 24 h urine and feces. The day after the last dose, the rats were euthanized. Blood and femurs were harvested immediately for further analysis.

### 2.7. Sample Collection

During the experiment, body weight and the amounts of urine and feces were recorded daily. In the last two days, 24 urine and fecal samples were collected. After the 4-week treatment, all SD rats were fasted for 12 hours before being anesthetized and sacrificed and dissected to obtain blood and femur specimens. The residual muscle of the left and right femurs of each rat was cleaned. The left femoral bone samples were intended for bone densitometry, and samples of right femoral bone were stored in 4% paraformaldehyde for histopathological analysis.

### 2.8. Alkaline Phosphatase (ALP), Serum Calcium, and Serum Phosphorus (P) Expression Assay

Rat blood samples were centrifuged at 3500 rpm for 10 min to obtain the serum. The ALP content in the serum, serum calcium, and serum P were determined. ALP was quantitatively assayed using an ALP detection kit (Nanjing Jiancheng Bioengineering Co., Ltd., China). Serum calcium and serum phosphorus were measured using kits (Nanjing Jiancheng Bioengineering Co., Ltd., China) according to a protocol specified by the manufacturer. Serum calcium was measured through the methyl thymol blue method. The OD was determined at a wavelength of 610 nm.

### 2.9. Calcium Balance Study

The calcium content of feces and urine samples from SD rats was determined [16]. The following equations were used to calculate the calcium absorption rate and calcium balance:

Ca absorption rate (%) = (Ca intake − fecal Ca)/Ca intake × 100 (1).

Ca balance = Ca intake − (urinary Ca + fecal Ca) (2).

### 2.10. Femur Condition Detection

The length and wet weight of the bilateral femur bones were recorded. The left femurs were calcined in a muffle furnace at 600°C for 8 h, and the remaining weight was recorded as the dry weight. BMD was measured by using dual-energy X-ray absorptiometry with a bone densitometer (GE Healthcare, USA).

### 2.11. Bone Mineralization Assay

A calibration standard solution of calcium (GSB 04-1720-2004, National Nonferrous Metals and Electronic Materials Analysis and Testing Center, China) was used. HCl (1 ml, 6 mol/L) was added to dissolve the ashes (0.1 g) of the left femur. Then, a 10 µl solution of the left femur was used to detect the calcium content. The phosphorus content in the femurs was detected by inorganic calcium and phosphorus assay kits (Jiancheng Bioengineering Co., Ltd., China).

### 2.12. Histopathology Observation

Thigh bone weight was weighed, measured, and conventionally fixed in 10% formalin at 4°C overnight. All femur samples were decalcified for 24 h in an 8% formic acid/hydrochloric acid solution. After the brief water wash, femurs were transferred to ammonia solution to neutralize acids and left to stand for 30 min. Tissues were processed in an automated tissue processor for dehydration and clearing. After dehydration in an ethanol series, the samples were embedded in paraffin, and sections (4 µm) were made. Strips of tissue were stained with hematoxylin and eosin (HE) for morphological evaluation. Then, all the sections were dehydrated with graded ethanol and xylene. The slides (*n* = 10) were examined under a light microscope (OlympusCorp., Tokyo, Japan) using 400× magnification.

### 2.13. Statistical Analysis

Statistical analyses were performed by using the SPSS 8.0 software. Data are expressed as the means ± S.D. The level of significance was set at *p* < 0.05.

## 3. Results and Discussion

### 3.1. The Fabrication of FECa

**Figure 1:**
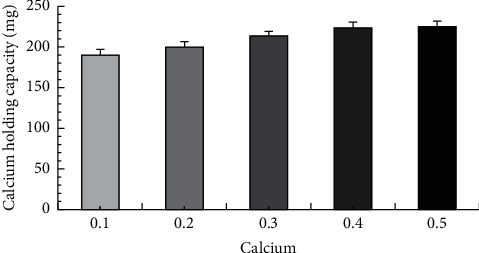
Calcium holding capacity of FECa.

Different amounts of CaCO_3_ were added to the FE solution to obtain the optimal synthesis ratio of FE and calcium ions. The increase in the calcium holding capacity of FECa is observed with increasing CaCO_3_ amount, and the calcium holding capacity of FECa reaches 16.85% (shown in Figure 1) when the addition of CaCO_3_ is 0.4 g. No significant changes are shown in the calcium holding capacity of FECa (*p* > 0.05) when continuing to increase the amount of CaCO_3_.

### 3.2. Morphological Analysis of FECa

SEM and EDS were conducted to determine the morphology and elemental composition of FECa [17]. Compared with FE, the morphology of FECa is rougher with many irregular particles.

**Figure 2:**
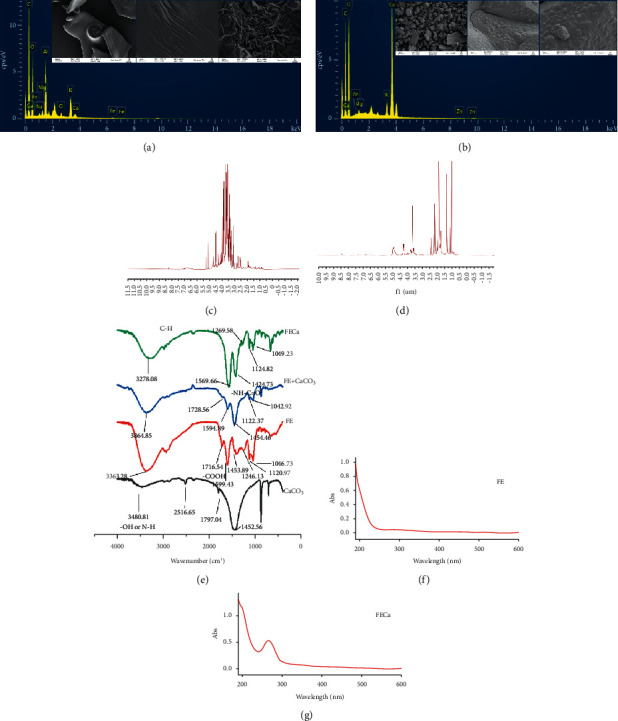
Microstructure and spectroscopy analysis of FE and FECa. SEM (200×, 10 k×, scale bar = 2 *μ*m) and EDS mappings of FE (a) and FECa (b), ^1^H-NMR of FE (c) and FECa (d), FTIR spectroscopy (e), and UV spectroscopy (f) and (g).

The composition and distribution of elements of FE and FECa were detected by EDS. The EDS spectra of FE and FECa (shown in Figure 2(a) and 2(b)) show that the contents of oxygen (O) and calcium in FE are different from those in FECa. Oxygen changes little, and calcium changes greatly, which may be due to the hydrogen in calcium and hydroxyl groups. Combined with the figure, it can be seen that looking at the appearance of the sample and the composition of surface elements, it is found that the differences in oxygen and calcium content between FE and FECa corresponding with the SEM results showed that the fermented *R. roxburghii* Tratt had a coupling reaction with CaCO_3._

### 3.3. FTIR, ^1^H-NMR, and UV Spectroscopy

The FTIR, ^1^H-NMR, and UV spectroscopy of FE and FECa were recorded to determine the binding site of calcium ions [17–20].

The wavenumbers of FE, FECa, FE + CaCO_3_, and CaCO_3_ were recorded from 4000 to 400 cm^−1^. The spectra of FE and FE + CaCO_3_ are largely similar, while those of FE and FECa exhibit some differences (shown in Figure 2(e)). The strong absorption band at approximately 3400 cm^−1^, corresponding to the stretching of –OH and N-H, is observed in the FTIR spectra analysis of FE and FECa. The spectral band at approximately 2930 cm^−1^ represents the C-H stretching vibration. The spectral band at 1560 cm^−1^ includes C=O stretching, vibrations specific to C-N stretching, and N-H deformation, which indicate the –NH–C=O group. The absorption band at 1680–1740 cm^−1^ represents unionized carboxyl groups, while 1550–1620 cm^−1^ represents ionized carboxyl groups. The unionized carboxyl group absorption band of FE is at 1716 cm^−1^, while that of FECa disappears. In addition, the absorption band of FECa at 1569 cm^−1^ is strengthened, which indicates that calcium could bind the ionized carboxyl group of FE [6, 21, 22].

The UV spectra of FE and FECa are shown in Figures 2(f) and 2(g). The maximum absorption peak of FECa is at 265 nm, but that of FE is not observed, which may be assigned to the changes in the FE constituents induced by the introduction of calcium ions.

The ^1^H-NMR spectra of FE and FECa are shown in Figures 2(c) and 2(d). FECa is different from FE. The NMR spectra of FE revealed the presence of obvious signals at *δ*H 3.5–4.2 and 6.3–8.1, according to our previous work in constituents of FE, which might represent some saccharide, flavonoids, and aromatic signals, respectively, while signals of FECa in these two regions are reduced. The overlapping hydrogen signals of FECa at *δ*_H_ 1.20–2.40 strengthen, which might indicate that some small molecules, such as organic acids, in FECa are different.

### 3.4. Body Weight and Biochemical Parameters in Serum

**Figure 3:**
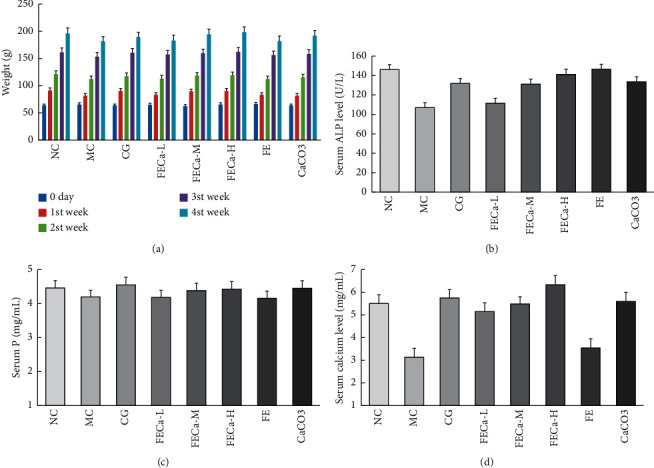
Body weight and biochemical parameters in serum. Body weight of SD rats (a), serum ALP (b), serum P (c), and serum calcium (d). NC: normal control group; MC: model control group; L: low dose of FECa; M: middle dose of FECa; H: high dose of FECa; CaCO_3_: CaCO_3_ group; CG: calcium gluconate group.

Further functions of FECa in promoting BMD were studied in low-calcium dietary SD rats. After oral administration (as shown in Figure 3(a)), the body weights of the MC group were lower than those of the NC group (*p* < 0.05). The middle- and high-dose groups were significantly higher than the MC group (*p* < 0.05).

Alkaline phosphatase (ALP) is a very well-known marker of bone anabolic activity and a key component of bone metabolism and is expressed early in the development of bone and calcification of cartilage tissues as well as on the cell surface of osteoblasts and in matrix vesicles [23, 24]. Decreased ALP activities (Figure 3(b)) in the serum may indicate that bone formation was inhibited while bone absorption was activated. In this study, ALP activity in the FECa-treated groups increased with increasing concentrations of FECa and showed a dose-response relationship, which indicated that the disrupted balance between bone formation and absorption was modified. In addition, ALP levels were higher in the FE group with respect to FECa-L, FECa-M, FECa-H, and CaCO_3_, possibly because some compounds only in FE improved the bioactivity of alkaline phosphatase.

Bone metabolism is an important aspect of skeletal development, homeostasis of serum calcium (Ca) and phosphate (P) levels, and maintenance of haematopoiesis [25–27]. As Figure 3(c) and 3(d) show that the serum *p* level of SD rats was not significantly changed (*p* < 0.05). However, the serum Ca level of SD rats fed FECa increased compared with the model group (*p* < 0.01).

### 3.5. Bone Biomechanical Parameters

**Figure 4:**
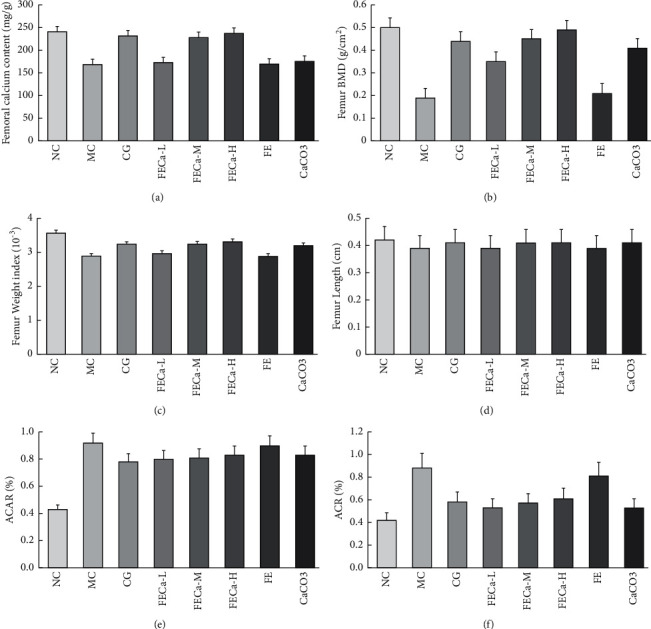
Bone biomechanical parameters and calcium balance. Femoral calcium content (a), femur BMD (b), femur weight index (c), femur length (d), fractional calcium absorption (ACAR) (e), and calcium retention (ACR) (f). NC: normal control group; MC: model control group; L: low dose of FECa; M: middle dose of FECa; H: high dose of FECa; CaCO_3_: CaCO_3_ group; CG: calcium gluconate group.

BMD is an important role of human bone strength and can affect the risk of osteoporosis along with other environmental factors [28]. The BMD of the MC group rats was greatly reduced compared with that of the NC group (*p* < 0.05), which means that the low-calcium model was established successfully (Figure 4(b)). Compared with rats in the MC group, the rats treated with FECa had a higher BMD, which explains the protective effect of FECa on promoting BMD. The dry weight analysis of the femurs (Figure 4(c)) also shows that FECa could convert the bone weight decrease. The femur lengths of the NC group were slightly lengthened than those of the MC group (*p* < 0.05), while there was no significant difference in CG, CaCO_3_, FECa-M, and FECa-H (*p* < 0.05).

Compared with the MC group, the calcium contents of the femurs in the high-dose group rats were greatly increased by approximately 36% (Figure 4(a), *p* < 0.01). These results are consistent with our previous data. Specifically, animals were subjected to a calcium balance study [29–31]. Fractional calcium absorption (ACAR) and calcium retention (ACR) were calculated based on the results from urine and fecal outputs. A higher ACAR in the MC group rats was observed. Generally, when the calcium concentration in the blood is lowered, the secretion of parathyroid hormone (PTH) is increased, which then increases the which then increases the reabsorption of calcium in the kidneys [32]. Compared with the CG and CaCO_3_ group, the FECa-M group showed increased ACAR and ACR. Based on the experiments described above, we concluded that FECa could enhance calcium balance in SD rats.

### 3.6. Femur Hematoxylin-Eosin Staining (H&E) and Computed Tomography (CT) Scan

**Figure 5:**
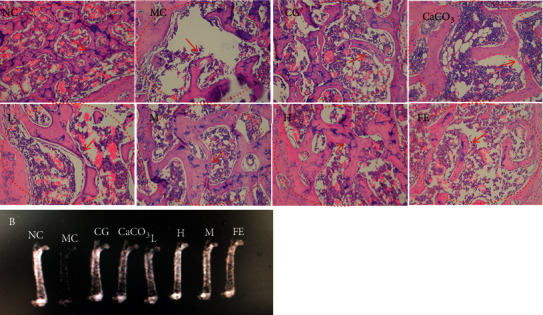
Femur H&E staining and CT scan study of SD rats treated with FECa. NC: normal control group; MC: model control group; L: low dose of FECa; M: middle dose of FECa; H: high dose of FECa; CaCO_3_: CaCO_3_ group; CG: calcium gluconate group.

Femur H&E staining and CT scans of SD rats after administration of FECa were performed. The results (Figure 5) show that the MC group rats had fewer scattered bone trabeculae, while obviously wider and more trabeculae in the FECa groups were observed in a dose-dependent manner. The bone trabeculae of the NC group were intact, regularly arranged and compacted, while trabeculae of the MC group were sparse, thinned, and even broken; the structure was disordered and some fragments appeared. All mentioned situations in the MC group were improved after oral administration of FECa. Additionally, sparse trabeculae of the MC group and compacted trabeculae of the FECa groups are shown in the CT scan.

## 4. Conclusion

The calcium holding capacity, morphology and elemental composition, characterization, and efficacy in promoting the BMD of FECa were examined in this study. Compared with the MC group, the calcium contents of the femurs were increased by 36%, and net calcium balance was enhanced in rats treated with FECa. Using H&E staining of the femur and CT scans of SD rats, it was shown that the width and number of trabeculae from the FECa groups were obviously enlarged, further indicating a dose-dependent effect. The above results indicated that FECa can be a potential and efficient calcium supplementation agent. The biomechanism of BMD in FECa should be investigated in our future work. Although good solubility and activity on bone mineral density from FECa organic calcium is obtained, there are limitations in this study, such as what kinds of chemical composition and how do they work. These limitations should be further explored. This study provides an idea for the preparation and characterization of calcium-incorporated fermented fruit and its efficacy on bone mineral density in rats [33].

## Data Availability

All the data are included in the article.
